# Machine learning algorithm to predict the in-hospital mortality in critically ill patients with chronic kidney disease

**DOI:** 10.1080/0886022X.2023.2212790

**Published:** 2023-05-19

**Authors:** Xunliang Li, Yuyu Zhu, Wenman Zhao, Rui Shi, Zhijuan Wang, Haifeng Pan, Deguang Wang

**Affiliations:** a Department of Nephrology, The Second Affiliated Hospital of Anhui Medical University, Hefei, People’s Republic of China; b Institute of Kidney Disease, Inflammation & Immunity Mediated Diseases, The Second Affiliated Hospital of Anhui Medical University, Hefei, People’s Republic of China; c Department of Epidemiology and Biostatistics, School of Public Health, Anhui Medical University, Hefei, People’s Republic of China; d Inflammation and Immune Mediated Diseases Laboratory of Anhui Province, Hefei, People’s Republic of China

**Keywords:** Chronic kidney disease, machine learning, mortality, intensive care unit, critically care

## Abstract

**Background:**

This study aimed to establish and validate a machine learning (ML) model for predicting in-hospital mortality in critically ill patients with chronic kidney disease (CKD).

**Methods:**

This study collected data on CKD patients from 2008 to 2019 using the Medical Information Mart for Intensive Care IV. Six ML approaches were used to build the model. Accuracy and area under the curve (AUC) were used to choose the best model. In addition, the best model was interpreted using SHapley Additive exPlanations (SHAP) values.

**Results:**

There were 8527 CKD patients eligible for participation; the median age was 75.1 (interquartile range: 65.0–83.5) years, and 61.7% (5259/8527) were male. We developed six ML models with clinical variables as input factors. Among the six models developed, the eXtreme Gradient Boosting (XGBoost) model had the highest AUC, at 0.860. According to the SHAP values, the sequential organ failure assessment score, urine output, respiratory rate, and simplified acute physiology score II were the four most influential variables in the XGBoost model.

**Conclusions:**

In conclusion, we successfully developed and validated ML models for predicting mortality in critically ill patients with CKD. Among all ML models, the XGBoost model is the most effective ML model that can help clinicians accurately manage and implement early interventions, which may reduce mortality in critically ill CKD patients with a high risk of death.

## Background

Chronic kidney disease (CKD) has become a severe global health concern, with roughly 700 million individuals currently suffering from CKD [1]. CKD is one of the world’s top 10 leading causes of mortality, impacting around 15% of the adult population [2]. As the intensive care unit (ICU) population shifts, many patients have preexisting CKD [3,4]. In recent years, despite more medical resources devoted to treating CKD, the life expectancy of CKD patients remains much lower than that of the general population [5]. According to one study, the risk of death is three times greater for patients with CKD in the ICU than those without CKD [6]. Early identification of CKD patients at high risk for clinical deterioration is of great importance and may help to deliver proper care and optimize the use of limited resources [7]. Thus, the clinical practice may benefit from developing predictive models that can accurately predict an individual’s survival prognosis.

Machine learning (ML) algorithms may offer an opportunity to reduce the risk of death from CKD in critically ill patients through their ability to analyze the vast amount of data in electronic health records. These data may include patient diagnoses, demographics, routinely obtained measures, and therapies. These cutting-edge data-driven methods can handle data with a high dimension, analyze complex relationships, and isolate essential predictors of outcomes. They are more flexible than traditional modeling techniques, which require predictors to be independent of each other and use variables selected primarily based on the statistical significance or clinical importance [8,9]. In recent years, ML methods have been widely used in the prognostic assessment of diseases [10–12]. Clinicians may better screen for and identify patients at high risk of adverse outcomes with a well-built prediction model, allowing for more prompt intervention and better outcomes. Unfortunately, no ML model can predict in-hospital mortality among critically ill CKD patients. This research aimed to establish and validate an ML model for predicting in-hospital mortality in critically ill patients with CKD.

## Methods

### Database introduction

The Medical Information Mart for Intensive Care IV (MIMIC IV) database is a comprehensive, anonymized clinical dataset approved by the Massachusetts Institute of Technology [13]. The MIMIC IV database contains data on all Beth Israel Deaconess Medical Center ICU patients between 2008 and 2019. As all patients in the database are anonymous and have no impact on clinical decision-making, the requirement for patient consent and ethically informed consent declarations was waived [14]. One author (XL) passed the Protecting Human Research Participants exam of the National Institutes of Health (record ID: 35970146) and gained permissible access to the MIMIC IV database.

### Study population

This research comprised all patients diagnosed with CKD who were enrolled in MIMIC IV. The diagnosis of CKD was based on the International Classification of Diseases, Ninth Revision (ICD-9) codes (5851, 5852, 5853, 5854, 5855, 5856, 5859), and International Classification of Diseases, Tenth Revision (ICD-10) codes (N18, N181, N182, N183, N184, N185, N186, and N189), which were recorded by hospital staff at the time of patient discharge. Patients admitted to the ICU more than once had their first admission counted. We eliminated patients under 18 and those who spent less than 24 h in the ICU.

### Data collection

This study identified candidate variables for the model based on clinical expertise and previous studies [1]. We used Navicat Premium to extract the demographic and clinical data from the MIMIC IV database. The research gathered age, gender, weight, ethnicity, and admission type as demographic factors. Medical conditions included congestive heart failure, peptic ulcer disease, myocardial infarction, peripheral vascular disease, diabetes, dementia, chronic pulmonary disease, rheumatic disease, cerebrovascular disease, cancer, paraplegia, liver disease, and acquired immune deficiency syndrome. Vital signs data, including heart rate, mean arterial pressure, respiratory rate, temperature, and oxygen saturation are averaged over the first 24 h after admission to the ICU. Laboratory results included hematocrit, hemoglobin, platelets, white blood cell, blood urea nitrogen, anion gap, international normalized ratio, serum creatinine, serum glucose, serum calcium, serum chloride, bicarbonate, serum potassium, serum sodium, partial thromboplastin time, and prothrombin time, all of which were maximum values within 24 h of admission to the ICU. The urine volume is recorded as the total value of the first 24 h after admission to the ICU. In addition, we recorded medical treatments such as renal replacement therapy, vasopressor use, and mechanical ventilation during the first 24 h following ICU admission. During the first 24 h following ICU admission, we determined the sequential organ failure assessment (SOFA) score and the first value of the simplified acute physiology score II (SAPS II) to use as the severity scores of illness. We also collected the patients’ CKD stage and estimated glomerular filtration rate (eGFR). Comorbidities for this study were defined according to the ICD-9 codes and ICD-10 codes [15].

### Endpoints

The endpoint of this study was in-hospital mortality.

### Preprocessing of data

There were less than 20% missing values for any variable in this study (Supplementary Table S1). The multiple interpolation methods are better for dealing with missing data below 20%. The multiple interpolation methods allow for the creation of multiple reasonable, fully interpolated datasets, which are first analyzed individually, and then their results are combined into a single result. We created 20 fully interpolated datasets using Python’s ‘micforest’ package and then pooled with Rubin’s rules. The estimated parameters were then pooled with Rubin’s rules [16].

### Statistical analysis

Continuous variables in this study were expressed as the median and interquartile range (IQR), and the Mann–Whitney test was used to determine differences between groups due to their non-normal distribution. Categorical variables were expressed as numbers and percentages, and group comparisons were made using the Chi-square test or Fisher’s exact test, as appropriate.

Statistical analyses were performed using R software (version 4.2.1) (R Foundation for Statistical Computing, Vienna, Austria) and Python (version 3.9.12). A *p* value <.05 was considered to be statistically significant.

### Machine learning

This study performed a hierarchical fivefold cross-validation to obtain the training set and validation sets. The study population was randomly divided into five subsets. Four of these subsets (80%) were combined as the training set, while the remaining (20%) were made the validation set, and this process was repeated five times for each outcome.

This study uses six ML techniques, including logistic regression, support vector machine (SVM), *k*-nearest neighbor (KNN), decision tree, random forest (RF), and eXtreme Gradient Boosting (XGBoost), to develop and validate models for the risk of death in critically ill patients with CKD. Logistic regression is a classification model. We have chosen the dichotomy logistic regression vs. ML because the logistic regression does not require the optimization of any hyperparameter and is thus easier to implement. SVM is a binary linear classifier. SVM separates different classes by establishing a decision boundary between two classes and optimizing the hyperplane distance between the boundary points, which can be obtained with reasonable accuracy from small data sets to achieve labeled prediction of one or more feature vectors. KNN is one of the most basic and simple ML algorithms. It can be used for both classification and regression. KNN performs classification by measuring the distances between different feature values. The decision tree is a single base classifier consisting of nodes and edges. Starting from the root node, also known as the first split point, the split determines the divisions of the entire dataset based on calculation. The process continues from top to bottom until no more partitioning is required, and the leaves present at the end of the decision tree represent the last partitions. RF is an ensemble learning method to overcome the drawbacks of a single base prediction model, aiming to achieve higher accuracy. This model includes multiple decision trees corresponding to various sub-datasets created from an identical dataset. XGBoost establishes K regression trees to make the predicted value of the tree group close to the real value as much as possible and can generalize as much as possible. The objective function of XGBoost requires the prediction error to be as small as possible, the number of leaf nodes to be as small as possible, and the number of nodes to be as low as possible.

We let each ML algorithm’s default hyper-parameters take effect to get started with a model. Afterward, we fine-tuned the parameters by searching the grid by hand. Tenfold cross-validation was used to find the optimal settings of the hyperparameters. The predictive performance of ML models was evaluated using accuracy, area under the curve (AUC), sensitivity, and specificity. For AUC, the 95% CI was computed with 2000 stratified bootstrap replicates. The testing AUC values corresponding to the different models were compared using paired Delong’s test. Accuracy and AUC were used to choose the best model. The probability of the best-performing model is evaluated using Brier scores and plotting the calibration curve for each model. To compare the predictive power between models, we calculated the Brier score for each model and plotted the calibration curve. Net reclassification improvement (NRI) was used to assess the correct reassignment between risk categories. In addition, the best model was interpreted using SHapley Additive exPlanations (SHAP) values and Local Interpretable Model-Agnostic Explanations (LIME) algorithm. Finally, a sensitivity analysis of the results was performed.

### Class imbalance

The in-hospital mortality rate of CKD patients in this study was 16.5%. As the performance of the ML model may be affected by class imbalance, we performed a complementary analysis using an up-sampling approach.

## Results

### Participants

**Figure 1.**
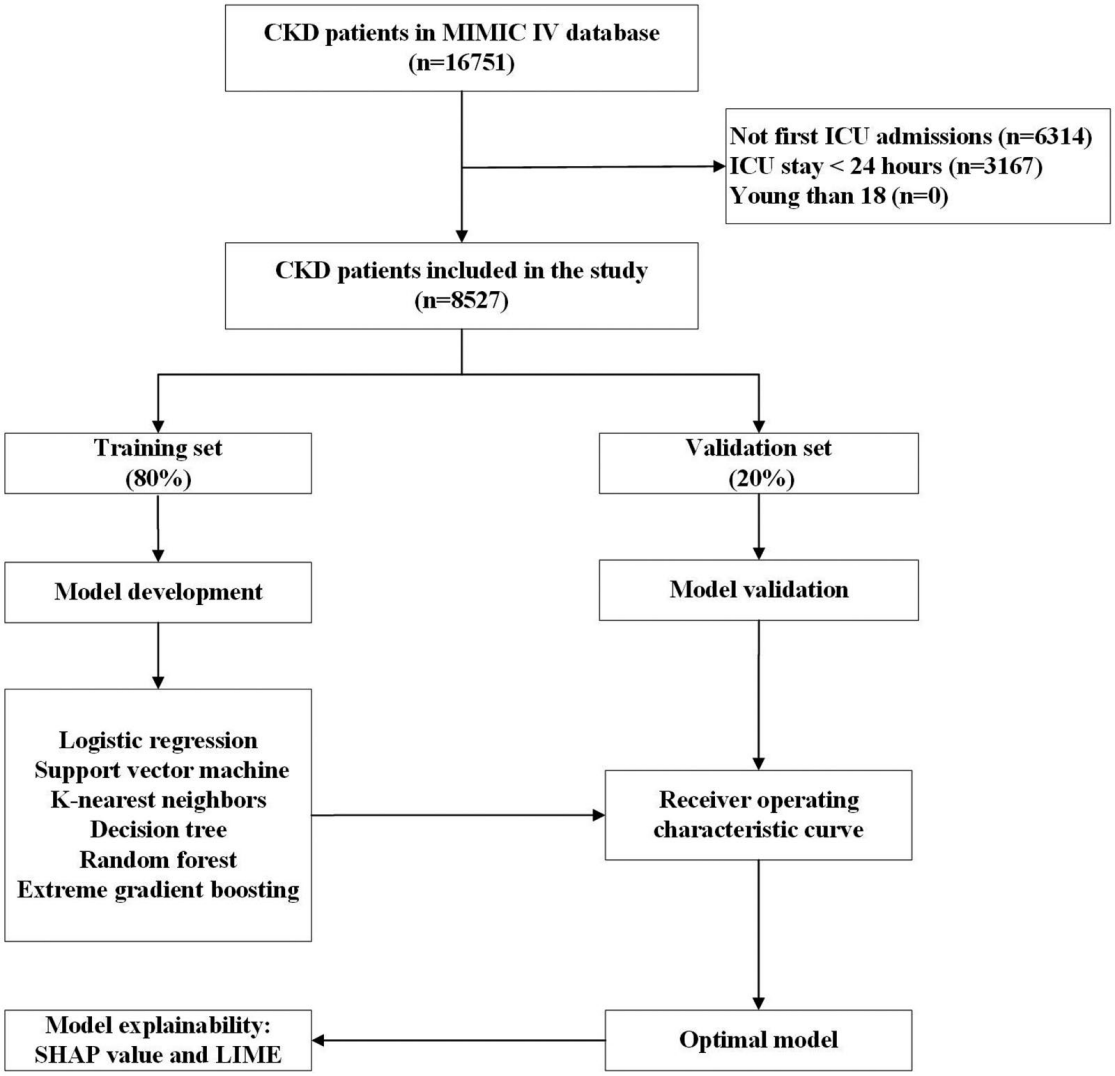
The flowchart of patient selection. MIMIC IV: Medical Information Mort for Intensive Care IV; ICU: intensive care unit; CKD: chronic kidney disease; LIME: Local Interpretable Model-Agnostic Explanations.

A total of 16,751 individuals were found to have CKD and be eligible to participate; however, 6314 were disqualified for non-first ICU admissions, and 3167 were disqualified due to having an ICU stay of fewer than 24 h. In the end, 8527 patients were eligible for the study (Figure 1). Among ICU-admitted CKD patients, the in-hospital death rate was 16.5% (1406/8527). The median age of these patients was 75.1 (IQR: 65.0–83.5) years, and 61.7% (5259/8527) were male. Congestive heart failure (4543/8527, 53.3%), diabetes mellitus (4222/8527, 49.5%), and sepsis (3516/8527, 41.2%) were the top three comorbidities. Table 1 provides a summary of the basic characteristics of the data set.

**Table 1.** Demographic and clinical characteristics at baseline.

Variables	Total (*n* = 8527)	Survivors (*n* = 7121)	Non-survivors (*n* = 1406)	*p* Value
*Demographics*				
Age (years)	75.1 [65.0, 83.5]	74.5 [64.4, 83.1]	77.5 [68.2, 85.4]	<.001
Sex, male, *n* (%)	5259 (61.7)	4416 (62.0)	843 (60.0)	.156
Weight (kg)	79.1 [66.9, 93.8]	79.6 [67.1, 94.0]	76.8 [65.0, 92.0]	<.001
Ethnicity, *n* (%)				<.001
White	5673 (66.5)	4759 (66.8)	914 (65.0)	
Black	1197 (14.0)	1033 (14.5)	164 (11.7)	
Others	1657 (19.4)	1329 (18.7)	328 (23.3)	
Admission type, *n* (%)				<.001
Urgent	1808 (21.2)	1442 (20.3)	366 (26.0)	
Emergency	6418 (75.3)	5399 (75.8)	1019 (72.5)	
Elective	301 (3.5)	280 (3.9)	21 (1.5)	
*CKD stage, n (%)*				<.001
Stage 1	151 (1.8)	132 (1.9)	19 (1.4)	
Stage 2	755 (8.9)	664 (9.3)	91 (6.5)	
Stage 3a	1538 (18.0)	1350 (19.0)	188 (13.4)	
Stage 3b	2356 (27.6)	2005 (28.2)	351 (25.0)	
Stage 4	2101 (24.6)	1641 (23.0)	460 (32.7)	
Stage 5	1626 (19.1)	1329 (18.7)	297 (21.1)	
*Comorbidities, n (%)*				
Myocardial infarction	2354 (27.6)	1917 (26.9)	437 (31.1)	.002
Congestive heart failure	4542 (53.3)	3688 (51.8)	854 (60.7)	<.001
Peripheral vascular disease	1647 (19.3)	1347 (18.9)	300 (21.3)	.039
Cerebrovascular disease	1289 (15.1)	1009 (14.2)	280 (19.9)	<.001
Dementia	467 (5.5)	378 (5.3)	89 (6.3)	.140
Chronic pulmonary disease	2544 (29.8)	2080 (29.2)	464 (33.0)	.005
Rheumatic disease	375 (4.4)	315 (4.4)	60 (4.3)	.850
Peptic ulcer disease	308 (3.6)	254 (3.6)	54 (3.8)	.671
Liver disease	1019 (12.0)	736 (10.3)	283 (20.1)	<.001
Diabetes	4222 (49.5)	3552 (49.9)	670 (47.7)	.134
Paraplegia	352 (4.1)	262 (3.7)	90 (6.4)	<.001
Cancer	1180 (13.8)	879 (12.3)	301 (21.4)	<.001
AIDS	50 (0.6)	43 (0.6)	7 (0.5)	.776
Sepsis	3516 (41.2)	2742 (38.5)	774 (55.0)	<.001
*Vital signs*				
Heart rate (beats/minute)	81.2 [71.8, 92.2]	80.5 [71.5, 90.9]	86.7 [74.3, 99.2]	<.001
MAP (mmHg)	74.9 [68.7, 82.6]	75.5 [69.2, 83.5]	72.2 [65.9, 79.0]	<.001
Respiratory rate (beats/minute)	18.8 [16.7, 21.5]	18.6 [16.6, 21.1]	20.3 [17.6, 23.4]	<.001
Body temperature (°C)	36.7 [36.5, 37.0]	36.8 [36.5, 37.0]	36.7 [36.4, 37.0]	<.001
SpO_2_ (%)	97.14 [95.7, 98.4]	97.2 [95.8, 98.4]	97.1 [95.4, 98.5]	.118
*Biochemical indices*				
Hematocrit (%)	32.1 [28.7, 36.5]	32.2 [28.8, 36.5]	31.7 [28.4, 36.7]	.110
Hemoglobin (g/dL)	10.4 [9.2, 11.8]	10.4 [9.2, 11.9]	10.2 [8.9, 11.7]	<.001
Platelets (K/μL)	199 [148, 266]	200 [150, 264]	197 [134, 278]	.019
WBC (K/μL)	12.1 [8.8, 16.9]	11.8 [8.6, 16.3]	14.0 [10.0, 19.3]	<.001
Anion gap (mEq/L)	17.0 [14.0, 20.0]	17.0 [14.0, 20.0]	19.0 [16.0, 23.0]	<.001
Bicarbonate (mmol/L)	24.0 [21.0, 27.0]	24.0 [21.0, 27.0]	23.0 [20.0, 26.0]	<.001
BUN (mg/dL)	39.0 [26.0, 60.0]	37.0 [25.0, 57.0]	49.0 [34.0, 73.0]	<.001
Serum calcium (mg/dL)	8.70 [8.20, 9.10]	8.70 [8.20, 9.10]	8.60 [8.10, 9.20]	.757
Serum chloride (mEq/L)	105 [100, 109]	105 [101, 109]	104 [99, 109]	<.001
Serum creatinine (mg/dL)	2.00 [1.40, 3.40]	1.90 [1.40, 3.20]	2.50 [1.70, 3.90]	<.001
Serum glucose (mg/dL)	150 [119, 206]	147 [117, 200]	168 [129, 232]	<.001
Serum sodium (mEq/L)	140 [137, 142]	140 [137, 142]	140 [137, 143]	.053
Serum potassium (mEq/L)	4.70 [4.20, 5.30]	4.70 [4.20, 5.20]	4.80 [4.30, 5.40]	<.001
INR	1.30 [1.20, 1.70]	1.30 [1.20, 1.60]	1.50 [1.30, 2.20]	<.001
PT (s)	14.9 [12.9, 18.7]	14.6 [12.7, 17.9]	16.6 [13.9, 23.9]	<.001
PTT (s)	34.3 [29.1, 48.6]	33.5 [28.8, 46.0]	39.5 [31.3, 63.0]	<.001
*eGFR, mL/min/1.73 m^2^*	33.3 [18.7, 47.6]	34.4 [19.4, 48.2]	28.1 [17.1, 42.0]	<.001
*Urine output (mL)*	1300 [670, 2100]	1390 [800, 2190]	744 [250, 1426]	<.001
*Medical treatments, n (%)*				
RRT	1130 (13.3)	882 (12.4)	248 (17.6)	<.001
Vasopressors use	456 (5.3)	264 (3.7)	192 (13.7)	<.001
Mechanical ventilation	6801 (79.8)	5547 (77.9)	1254 (89.2)	<.001
*Severity scores of illness*				
SOFA score	6 [4, 9]	5 [3, 8]	9 [7, 12]	<.001
SAPS II	41 [34, 50]	39 [33, 47]	51 [42, 62]	<.001

CKD: chronic kidney disease; AIDS: acquired immune deficiency syndrome; MAP: mean arterial pressure; SpO_2_: oxygen saturation; WBC: white blood cell; BUN: blood urea nitrogen; INR: international normalized ratio; PT: prothrombin time; PTT: partial thromboplastin time; eGFR: estimated glomerular filtration rate; RRT: renal replacement therapy; SOFA: sequential organ failure assessment; SAPS II: simplified acute physiology score II.

### Model development and validation

**Figure 2.**
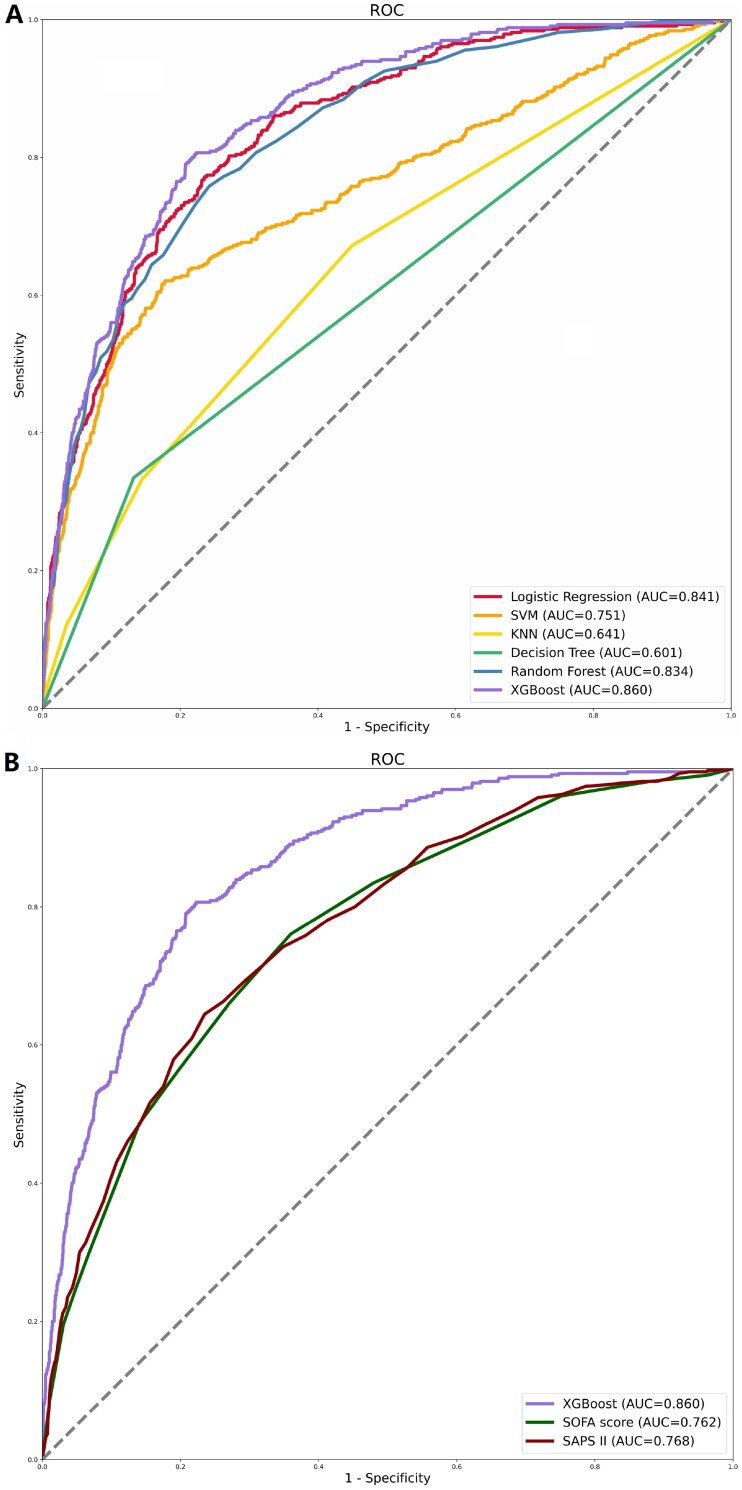
ROC curves for the ML models and the traditional severity of illness scores to predict in-hospital mortality. (A) ROC curves for the six ML models used to predict in-hospital mortality; (B) ROC curves for the traditional severity of disease scores used to predict in-hospital mortality. ROC: receiver operating characteristic; SVM: support vector machine; KNN; *k*-nearest neighbors; AUC: area under the curve; SOFA: sequential organ failure assessment; SAPS II: simplified acute physiology score II.

We developed six ML models using clinical variables as input factors, including logistic regression, SVM, KNN, decision tree, RF, and XGBoost. Compared to other ML models, the XGBoost model performed the best with an AUC of 0.860 (logistic regression: 0.841; SVM: 0.751; KNN: 0.641; decision tree: 0.601; RF: 0.834) (Figure 2(A)). The AUC in the XGBoost model was higher than in the other five models (*p* < .001) (Supplementary Table S2). Similarly, the XGBoost model outperformed various clinical disease severity scores (SOFA score (AUC): 0.762; SAPS II (AUC): 0.768) (Figure 2(B)). Table 2 displays the performance of our further analysis of the performance of these six ML models in terms of their precision, sensitivity, specificity, brier score, and NRI. Calibration plots for the six ML models are shown in Supplementary Figure S1.

**Table 2.** Performance comparison of the machine learning models in the testing set.

Models	Accuracy	AUC (95% CI)	Sensitivity	Specificity	Brier score	NRI
Logistic regression	0.838	0.841 (0.817–0.865)	0.772	0.765	0.045	0.000
SVM	0.832	0.751 (0.727–0.775)	0.621	0.822	0.054	0.001
KNN	0.823	0.641 (0.610–0.671)	0.672	0.550	0.085	0.002
Decision tree	0.787	0.601 (0.571–0.632)	0.335	0.868	0.160	−0.102
Random forest	0.855	0.834 (0.809–0.858)	0.758	0.758	0.051	0.157
XGBoost	0.860	0.860 (0.837–0.883)	0.807	0.777	0.036	0.211

AUC: area under the curve; CI: confidence interval; NRI: net reclassification improvement; SVM: support vector machine; KNN; *k*-nearest neighbors; XGBoost: eXtreme Gradient Boosting.

The brier score of the null model in this study was 0.165.

### Model explainability

**Figure 3.**
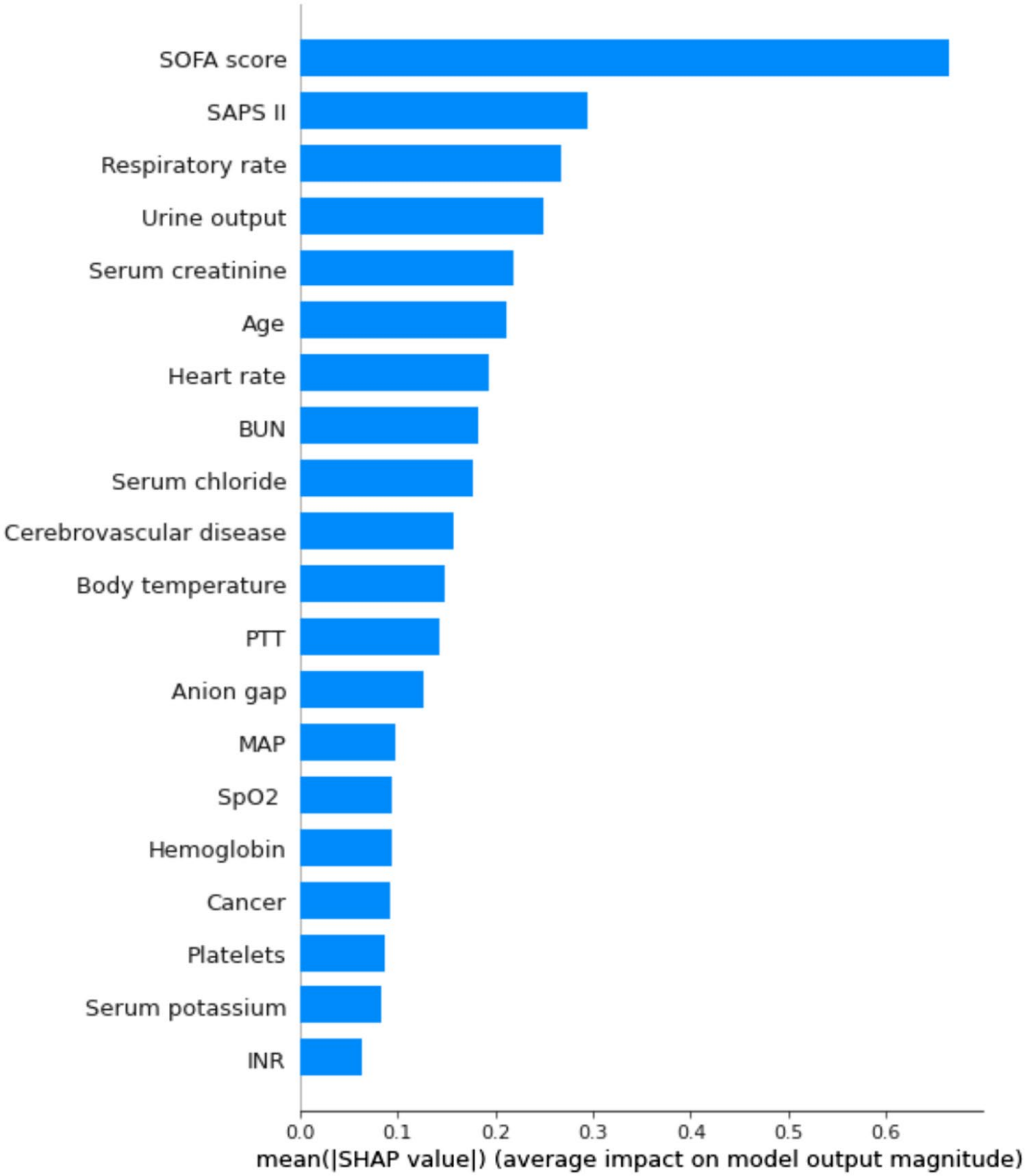
The top 20 important features derived from the XGBoost model. SHAP indicates the importance of ranking features. Each line represents a feature, and the abscissa is the SHAP value. The matrix plot represents the significance of each covariate in constructing the final predictive model. The higher the SHAP value for each clinical variable, the higher risk of death. SHAP: SHapley Additive exPlanations; SOFA: sequential organ failure assessment; SAPS II: simplified acute physiology score II; BUN: blood urea nitrogen; SpO_2_: oxygen saturation; MAP: mean arterial pressure; PTT: partial thromboplastin time.

**Figure 4.**
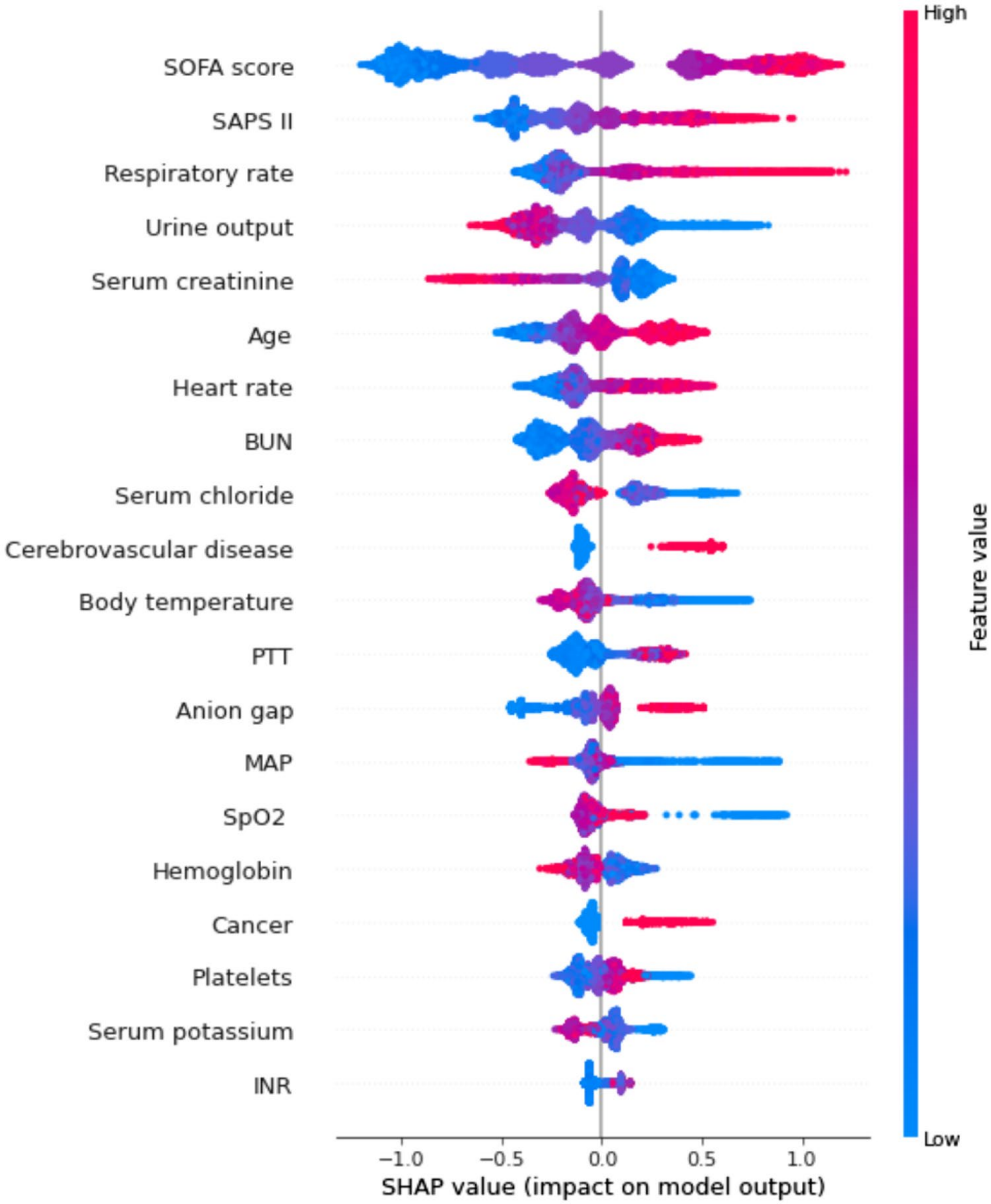
SHAP summary plot of the top 20 features of the XGBoost model. The importance matrix plot of clinical variables is derived using the XGBoost model. The matrix plot ranks the importance of the variables, revealing the contribution of each variable to death vs. survive. The greater the SHAP value of a characteristic, the greater the likelihood of death development. The abscissa represents the SHAP value, and each line represents a feature. Red dots indicate greater feature values, whereas blue dots indicate lower feature values. SHAP: SHapley Additive exPlanations; SOFA: sequential organ failure assessment; SAPS II: simplified acute physiology score II; BUN: blood urea nitrogen; SpO_2_: oxygen saturation; MAP: mean arterial pressure; PTT: partial thromboplastin time.

**Figure 5.**
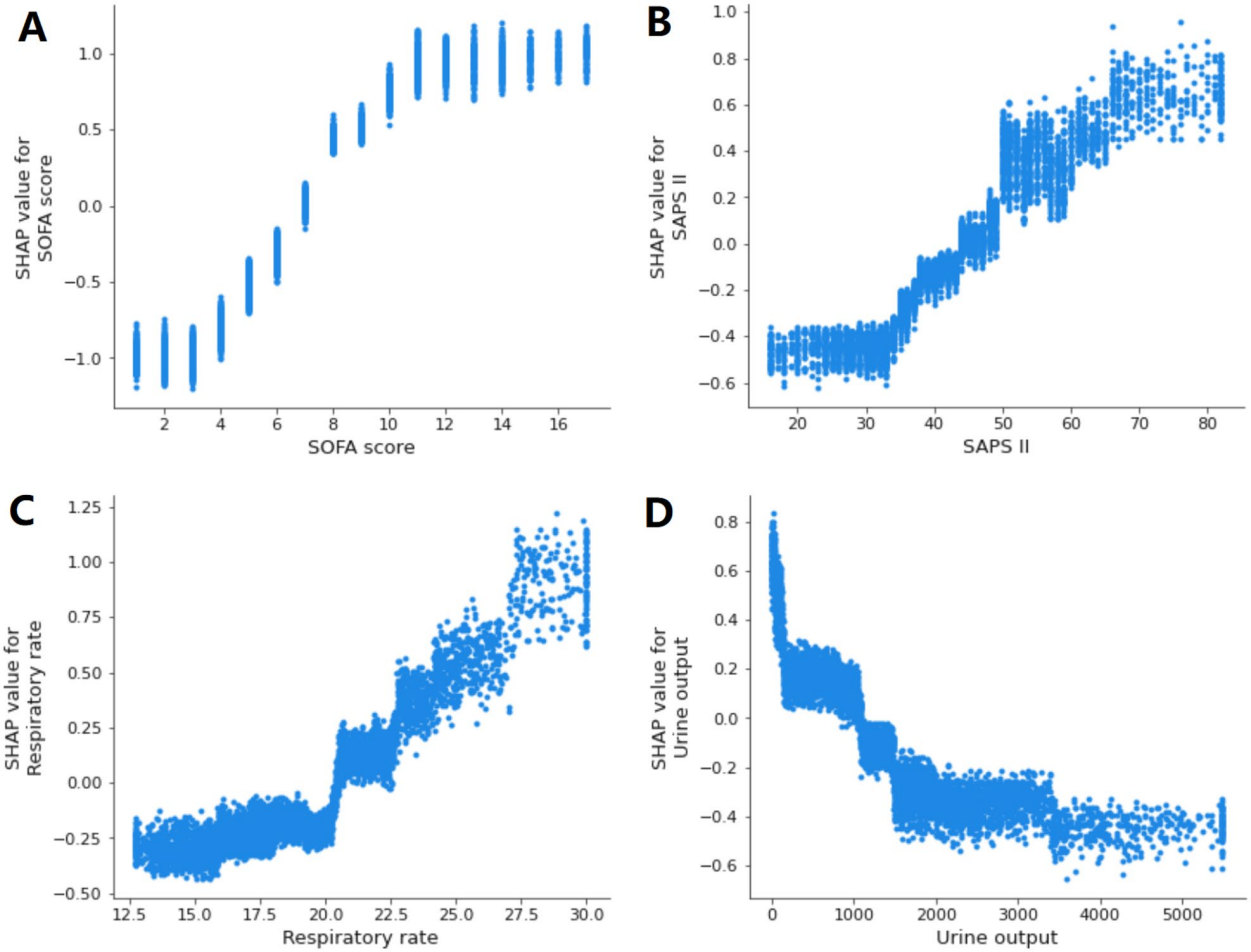
SHAP dependence plot of the XGBoost model. (A) SOFA score; (B) SAPS II; (C) respiratory rate; (D) urine output. SHAP values for specific features exceed zero, representing an increased risk of death. The greater the SHAP value of a characteristic, the greater the likelihood of death development. SHAP: SHapley Additive exPlanations; SOFA: sequential organ failure assessment; SAPS II: simplified acute physiology score II.

By using SHAP values, we aimed to elucidate the mortality prediction process of the XGBoost model. Figure 3 depicts the feature importance ranking of the XGBoost model with SHAP summary plots, where SOFA score, SAPS II, respiratory rate, and urine output are the four factors that contribute most to the model. In addition, we used SHAP dependence analysis to illustrate the effect of a single input variable on the final results of the XGBoost prediction model (Figure 4). Figure 5 shows the results of a more in-depth analysis of the four most influential clinical characteristics of the XGBoost prediction model output. In addition, we used the LIME algorithm to explain the individualized prediction of death by taking two samples (one survival and one deceased) from the validation set (Supplementary Figure S2).

### Sensitivity analyses

For patients with non-first ICU admission (*N* = 6314), the XGBoost model remained robust in predicting mortality in these patients (AUC: 0.821). Detailed results are shown in Supplementary Figure S3.

### Class imbalance

The performance results of the up-sampling approach show very similar results (Supplementary Table S3).

## Discussion

In this investigation, we constructed and tested six ML models for predicting in-hospital mortality in critically ill patients with CKD. The XGBoost model outperformed other models (including logistic regression, SVM, KNN, decision tree, and RF models) and traditional risk scores (including SOFA score and SAPS II) in predicting the death of critically ill patients with CKD. According to the feature importance evaluation, the four most important features of the XGBoost model that had the greatest predictive potential for mortality were the SOFA score, SAPS II, respiratory rate, and urine output. Moreover, we explain how these characteristics impact the XGBoost model. These results may contribute significantly to understanding ML models for predicting death in critically ill patients with CKD.

In recent years, CKD has profoundly impacted the prognosis and treatment options for several morbidities [17–19]. Furthermore, as the prevalence of CKD continues to rise in the general population and among ICU patients, preexisting CKD may drastically alter the treatment methods for these patients when admitted to the ICU [20–22]. Therefore, to identify those at high risk of clinical deterioration and facilitate early preventive measures that may reduce mortality, it is necessary to develop and promote prediction models that can early and swiftly predict death in critically ill patients with CKD.

In this analysis, the XGBoost model outperformed the other ML models in predicting mortality in CKD patients in critical care. The results of this study agree with those of numerous others. Liu et al. revealed that the XGBoost model outperformed other ML models, including logistic regression, RF, and SVM, in predicting death in acute kidney injury patients [23]. Hu et al. discovered that XGBoost performed better than SVM, KNN, logistic regression, decision tree, Naive Bayes, and RF [11]. A meta-analysis indicated that XGBoost outperformed other ML methods (such as SVM and Bayesian networks) for predicting acute kidney injury [24]. In addition, traditional severity scoring systems, such as the SOFA and SAPS II scores, performed poorly compared to ML models, indicating that they may not be reliable tools for predicting death in critically ill patients with CKD. In addition, our study showed that SOFA or SAPS II scores alone performed poorly in predicting mortality in critically ill patients with CKD compared with the ML model. Although the SOFA and SAPS II scoring systems may estimate the likelihood of bad outcomes in critically ill patients, excluding a significant number of relevant factors from their analyses may result in less accurate prediction than multivariable models [25]. Previous research has demonstrated that when compared to ML models, the SOFA score and SAPS II perform poorly in predictive performance [8].

In this investigation, we used the ML method for the first time to predict in-hospital mortality in critically ill patients with CKD. By ranking the importance of variables in the XGBoost model, we found that SOFA score, SAPS II, respiratory rate, and urine output were the variables that contributed most to predicting mortality in critically ill patients with CKD. The SOFA score is a tool that describes the presence of organ dysfunction [26]. It assigns each of the six organ systems (respiratory, circulatory, renal, hematologic, hepatic, and central nervous system) a daily score between 1 and 4 based on the severity of organ failure, with higher values indicating more severe organ dysfunction [27]. Some studies have shown that high SOFA scores are associated with higher mortality [28]. Similarly, the present research found that the SOFA score was the most significant predictor of death in critically ill patients with CKD, and it was given the highest weight in the XGBoost model. SAPS II is another essential factor that influences mortality. The SAPS II score comprises seventeen factors, with higher scores indicating illness severity [29]. Previous research has shown that SAPS II is related to a greater death rate among ICU patients [30]. In addition, we discovered that respiratory rate is a significant predictor of death in critically ill patients with CKD. Several studies have found an association between respiratory rate and worse outcomes [31]. Our research also showed a correlation between urine output and death among CKD patients in critical care. Oliguria is common in ICU patients and is the ultimate cause of renal parenchymal damage [32]. Some studies have shown that decreased urine output is associated with poor outcomes in critically ill patients [33].

However, this study also has some shortcomings. First, this was retrospective modeling research conducted at a single center using the MIMIC IV database, and we could not identify the causal association between characteristics and outcomes. In order to verify the accuracy of our approach, we need further prospective randomized clinical trials. Second, our research’s retrospective and observational design may inevitably result in selection bias. Third, we estimated specific missing data using padding, which may have led to discrepancies from the actual numbers. Finally, in this work, the model was only tested internally; external validation at multiple centers is needed to confirm the usefulness of the model.

## Conclusions

In conclusion, we successfully developed and validated ML models for predicting mortality in critically ill patients with CKD. Among all ML models, the XGBoost model is the most effective ML model that can help clinicians accurately manage and implement early interventions, which may reduce mortality in critically ill CKD patients with a high risk of death.

## Supplementary Material

Supplemental Material

## Data Availability

The datasets presented in the current study are available in the MIMIC-IV database (https://physionet.org/content/mimiciv/1.0/).
