# Supplementary material for: Machine learning algorithm to predict the in-hospital mortality in critically ill patients with chronic kidney disease
Source: Ren Fail. 2023 May 19;45(1):2212790. doi: 10.1080/0886022X.2023.2212790 (PMC10201999; doi:10.1080/0886022X.2023.2212790)
Supplement: Supplemental Material [file IRNF_A_2212790_SM9367.pdf]

Table S1. Missing number (%) for variables

Variables	Missing number (%)
**Demographic features**	
Age	0(0)
Sex	0(0)
Weight	346(4.1%)
Ethnicity	0(0)
Admission type	0(0)
**CKD stage**	21(0.3%)
**Chronic disease history**	
Myocardial infarction	0(0)
Congestive heart failure	0(0)
Peripheral vascular disease	0(0)
Cerebrovascular disease	0(0)
Dementia	0(0)
Chronic pulmonary disease	0(0)
Rheumatic disease	0(0)
Peptic ulcer disease	0(0)
Liver disease	0(0)
Diabetes	0(0)
Paraplegia	0(0)
Renal disease	0(0)
Cancer	0(0)
Aids	0(0)
Sepsis	0(0)
**Vital signs**	

Information Classification: General

Heart rate (beats/minute)	18(0.2%)
MAP (mmHg)	19(0.2%)
Respiratory rate (beats/minute)	22(0.3%)
Body temperature (°C)	302(3.5%)
SpO_2 (%)	18(0.2%)
**Biochemical indices**	
Hematocrit (%)	30(0.4%)
Hemoglobin (g/dL)	34(0.4%)
Platelets (K/uL)	33(0.4%)
WBC (K/uL)	33(0.4%)
Anion gap (mEq/L)	30(0.4%)
Bicarbonate (mmol/L)	26(0.3%)
BUN (mg/dL)	19(0.2%)
Serum calcium, (mg/dL)	522(6.1%)
Serum chloride, (mEq/l)	25(0.3%)
Serum creatinine (mg/dL)	21(0.3%)
Serum glucose (mg/dL)	41(0.5%)
Serum sodium (mEq/L)	26(0.3%)
Serum potassium (mEq/L)	28(0.3%)
INR	616(7.2%)
PT (s)	616(7.2%)
PTT (s)	666(7.8%)
**eGFR, ml/min/1.73 m2**	21(0.3%)
**Urine output**	610(7.2%)
**Treatments**	

Information Classification: General

RRT	0(0)
Vasopressors use	0(0)
Mechanical ventilation	0(0)
**Severity scores of illness**	
SOFA score	0(0)
SAPS II	0(0)

Abbreviations: CKD: chronic kidney disease, Aids: acquired immune deficiency syndrome, MAP: mean arterial pressure, SpO2: oxygen saturation, WBC: white blood cell, BUN: blood urea nitrogen, INR: international normalized ratio, PT: prothrombin time, PTT: partial thromboplastin time, eGFR: estimated glomerular filtration rate, RRT: renal replacement therapy, SOFA: sequential organ failure assessment, SAPS II: simplified acute physiology score II.

Information Classification: General

Table S2. Comparison of the AUCs between the six models using the DeLong method

	Logistic regression	SVM	KNN	Decision tree	Random forest	XGBoost
Logistic regression						
SVM	<0.001					
KNN	<0.001	<0.001				
Decision tree	<0.001	<0.001	<0.001			
Random forest	0.512	<0.001	<0.001	<0.001		
XGBoost	<0.001	<0.001	<0.001	<0.001	<0.001	

Abbreviations: AUC: area under the receiver operating characteristic curve, CI: confidence interval, SVM: support vector machine, KNN, k-nearest neighbors, XGBoost: Extreme Gradient Boosting.

Information Classification: General

Table S3. Comparison of AUC for machine learning models in the testing set using up-sampling

Models	AUC (95% CI)
Logistic regression	0.830 (0.806–0.854)
SVM	0.759 (0.735–0.783)
KNN	0.638 (0.607–0.668)
Decision tree	0.595 (0.564–0.627)
Random forest	0.838 (0.813–0.862)
XGBoost	0.855 (0.831–0.879)

Abbreviations: AUC: area under the receiver operating characteristic curve, CI: confidence interval, SVM: support vector machine, KNN, k-nearest neighbors, XGBoost: Extreme Gradient Boosting.

Information Classification: General

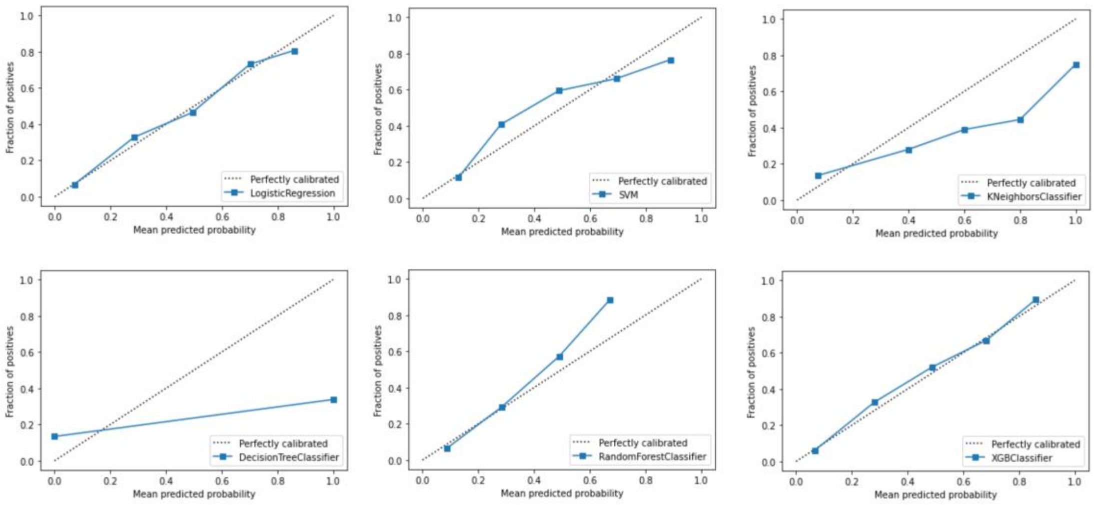

Fig. S1 The calibration curves for the six models

Abbreviations: SVM: support vector machine.

Information Classification: General

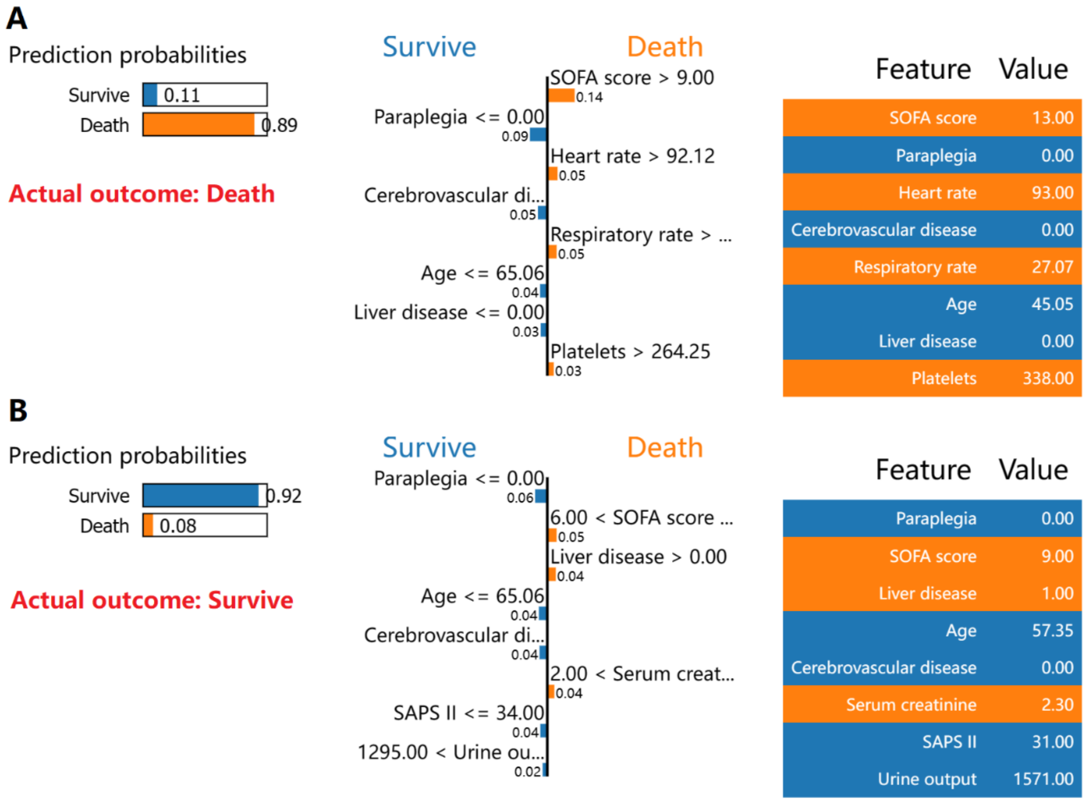

Fig. S2 Local Interpretable Model-Agnostic Explanations (LIME) algorithm for explaining individual's prediction results

(A) and (B) present a deceased case and a survival case with the LIME algorithm, respectively. The left part of the figure shows predicted results. The middle part presents the top 8 variables that had the greatest impact on survival or death from top to bottom, the length of the bar for each feature indicates the importance (weight) of that feature in making the prediction. A longer bar indicates a feature that contributes more towards survival or death. The right part shows the critical values of these 8 variables when they had the greatest impact on survival or death.

Abbreviations: SHAP: SHapley Additive explanation, SOFA: sequential organ failure assessment, SAPS II: simplified acute physiology score II.

Information Classification: General

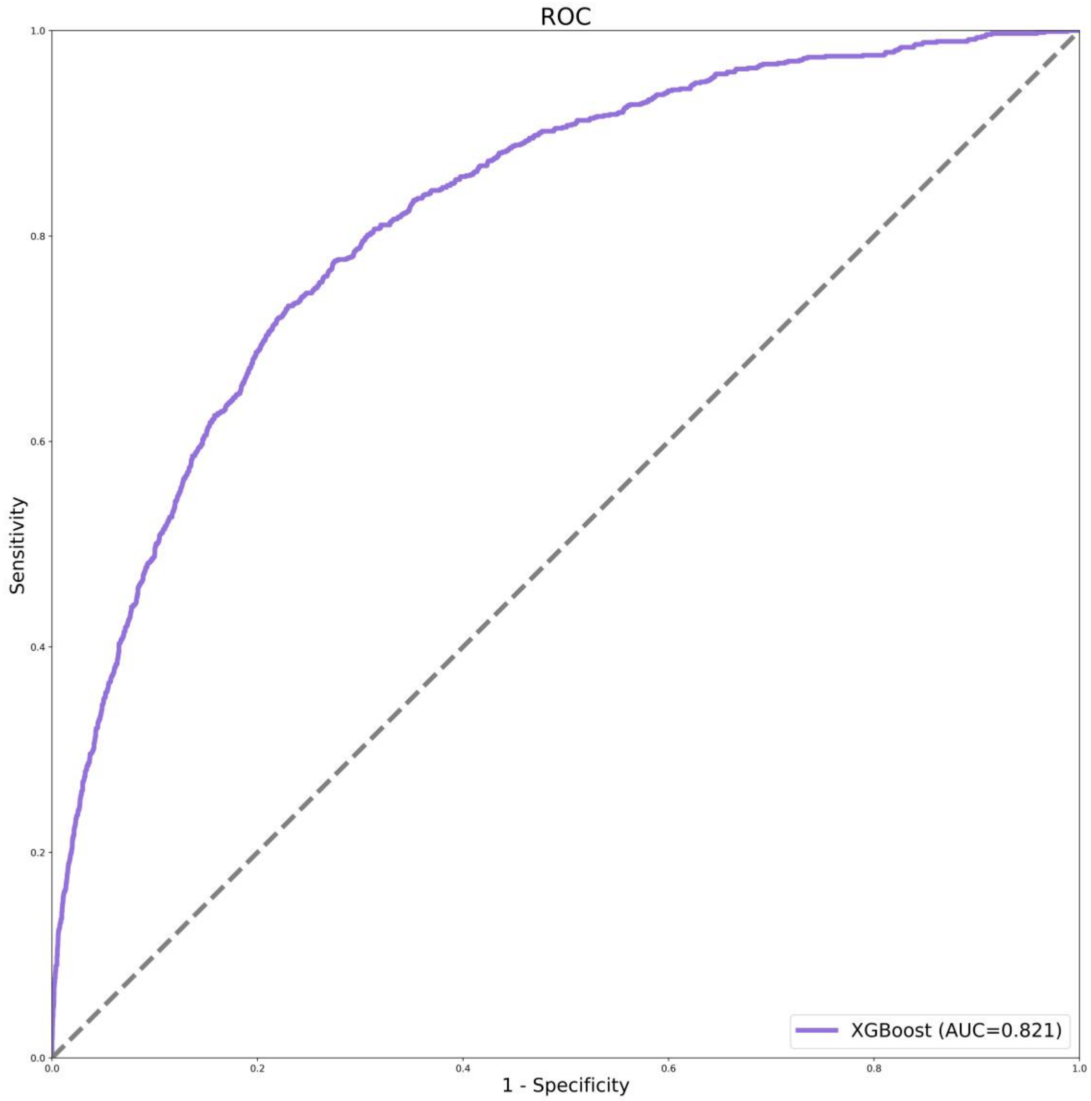

Fig. S3 ROC curves of XGBoost models for predicting in-hospital mortality in non-first ICU admissions.

Information Classification: General
